# Nanogenerator for determination of acoustic power in ultrasonic reactors

**DOI:** 10.1016/j.ultsonch.2021.105718

**Published:** 2021-08-16

**Authors:** Krystian Mistewicz, Marcin Jesionek, Hoe Joon Kim, Sugato Hajra, Mateusz Kozioł, Łukasz Chrobok, Xudong Wang

**Affiliations:** a Institute of Physics – Center for Science and Education, Silesian University of Technology, Krasińskiego 8, 40-019 Katowice, Poland; b Department of Robotics Engineering, Daegu Gyeongbuk Institute of Science & Technology (DGIST), Daegu 42988, South Korea; c Faculty of Materials Engineering and Metallurgy, Silesian University of Technology, Krasińskiego 8, 40-019 Katowice, Poland; d Department of Materials Science and Engineering, University of Wisconsin-Madison, Madison, WI 53706, USA

**Keywords:** Nanogenerator, Nanowires, Piezoelectric effect, Ultrasounds, Acoustic power

## Abstract

This paper presents the novel use of a sonochemical reaction product as a sensing material in self-powered ultrasonic reactor devices for determination of ultrasound parameters. A piezoelectric nanogenerator was fabricated via sonochemical synthesis of SbSeI nanowires compressed into a bulk sample. The prepared device was used to develop two fast and simple evaluation methods for acoustic power in liquid. A calibration procedure was carried out for both methods using a VCX-750 ultrasonic processor. The ultrasound acoustic power was varied within a 150 W to 750 W range and the corresponding nanogenerator electrical responses were measured. The voltage signals of the first method fit the best with theoretical dependence. The second technique was based on the application of the Fast Fourier Transform (FFT) to the measured electric output. The results of these two approaches were convergent. Acoustic power values of 255(8) W and 222(7) W were determined for the Sonic-6 reactor using theoretical dependence fitting to experimental data and FFT analysis, respectively. Developed sensing technology possesses great potential for sonochemistry applications.

## Highlights

• Sonochemical synthesis and high pressure processing used for nanogenerator fabrication.
• Two simple, and convergent methods of evaluation of acoustic power in liquid proposed.
• Nanodevice response best fitted with the theoretical dependence in the first method.
• The second technique based on Fast Fourier Transform analysis.
• The rapid measurement enables real time monitoring of ultrasonic reactor operation.

## Graphical abstract

**Figure:**
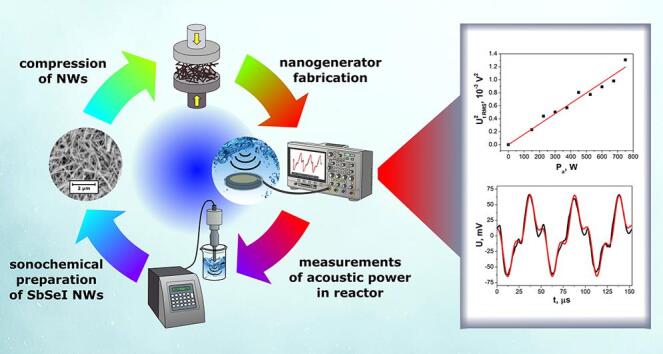

## 1 Introduction

Ultrasounds are widely used in industry, medical imaging, military technologies, and materials science. The measurement of acoustic energy in liquid is especially significant for characterization of different effects or phenomena occurring under ultrasound, e.g. cavitation, emulsification, erosion, sonoluminescence, and sonochemical reactions. Regarding sonochemical processes, this parameter can be used to estimate energetic yields [1], [2] as well as radical and molecular product formation rates [3]. Until now, many experimental techniques have been developed to evaluate acoustic power in fluids. Among them is the calorimetric method [2], [4], [5] which is based on the comparative measurement of liquid temperature change under heated ultrasound [3]. Another approach is the application of a polyvinylidene fluoride (PVDF) pyroelectric membrane sensor [6], [7], [8] which absorbs ultrasounds. The resultant increase in membrane temperature generates a voltage across the electrodes where the magnitude is proportional to the rate of temperature change with respect to time [7]. Hydrophones are most common devices used for liquid ultrasound characterization [9]. Hydrophone design can be divided into three groups: fiber-optic devices [10], [11], [12], piezoelectric ceramics [13], [14], [15], and membrane transducers [16], [17]. However, each hydrophone type possesses essential drawbacks. Fiber-optic hydrophones suffer from low sensitivity and working point drift induced by temperature or pressure dependent changes of cavity length [10]. The analysis of piezoelectric ceramic hydrophone electrical impedance response requires extensive knowledge of transducer parameters, including their physical dimensions, acoustic impedance, and the coupling factor [16]. Moving on to membrane devices, these transducers in particular are very susceptible to mechanical failure under high power ultrasounds [18]. In the last decade, low dimensional structures have gained prominence for harvesting different types of energy [19], [20], [21], [22]. Piezo- and triboelectric nanomaterials are particularly attractive for use in audible acoustic wave sensors [23], [24], [25], [26], [27] or ultrasounds [28], [29] as they can be operated without an external power source.

Antimony selenoiodide (SbSeI) is a semiconductor material with a relatively narrow energy band gap and thermoelectric [30], [31], ferroelectric [32], and piezoelectric properties [30], [33], [34], [35]. SbSeI is an excellent candidate for photovoltaic devices [36], [37], [38] and hard radiation detectors [39], [40]. Recently [41], a scalable and inexpensive SbSeI nanowire processing method has been developed for functional device applications. SbSeI nanowires are sonochemically grown and compressed under high pressure to form a macroscopic sample without the need for any thermal treatment. This simple processing was used to fabricate a SbSeI humidity nanosensor [42] and pyroelectric [41] nanogenerator. Furthermore, this method was also used to successfully prepare piezoelectric devices for mechanical energy harvesting [35]. SbSeI nanowires are remarkable materials for the detection of dynamic pressure changes and vibrations with low frequencies up to 200 Hz [35]. Despite the facile fabrication procedure of this device, its piezoelectric output is comparable to or better than much more sophisticated nanogenerators based on nanocomposites or other nanomaterial arrays [35]. However, the piezoelectric response of SbSeI nanowires has been examined only in the low frequency range, i.e. from 0.75 Hz to 200 Hz. Therefore, future investigation of ultrasonic excitation on the electric output of SbSeI nanogenerator is required.

This paper is the first report to present an application of sonochemically prepared nanomaterials in a self-powered device for determination of ultrasound parameters in an ultrasonic reactor. Additionally, the piezoelectric response of SbSeI nanowires for ultrasonic excitation has been examined or the first time. Two facile and accurate methods for acoustic power measurement have been developed. These methods can be successfully used for detailed analysis of ultrasonic waves emitted by a sonotrode or ultrasonic transducer. These techniques provide convenient manners for operation inspection of ultrasonic reactors.

## 2 Materials and methods

### 2.1 Material synthesis, its characterization, and nanogenerator fabrication

A standard sonochemical procedure was used to prepare SbSeI nanowires [35], [42]. The material was synthesized using antimony, selenium, and iodine subjected to ultrasonic irradiation. The reagents were weighed in a stoichiometric ratio and immersed in ethanol. This mixture was put into a plastic vessel and placed in a water bath in a VCX-750 ultrasonic reactor (Sonics & Materials, Inc.). Sonochemical preparation of SbSeI gel was carried out at 323 K within 2 h. SbSeI gel was then maintained at elevated temperature (313 K) for 10 h. Evaporation of the ethanol from the material resulted in a SbSeI xerogel.

The scanning electron microscopy (SEM) and energy dispersive X-ray spectroscopy (EDS) were used to examine the morphology and chemical composition of the prepared material. These studies were completed using a Phenom Pro X (Thermo Fisher Scientific) microscope integrated with EDS spectrometer. The acceleration voltage was adjusted to 10 kV and 15 kV for SEM and EDS surveys, respectively. The EDS results were quantified using ProSuite Element Identification (Thermo Fisher Scientific) software.

**Fig. 1:**
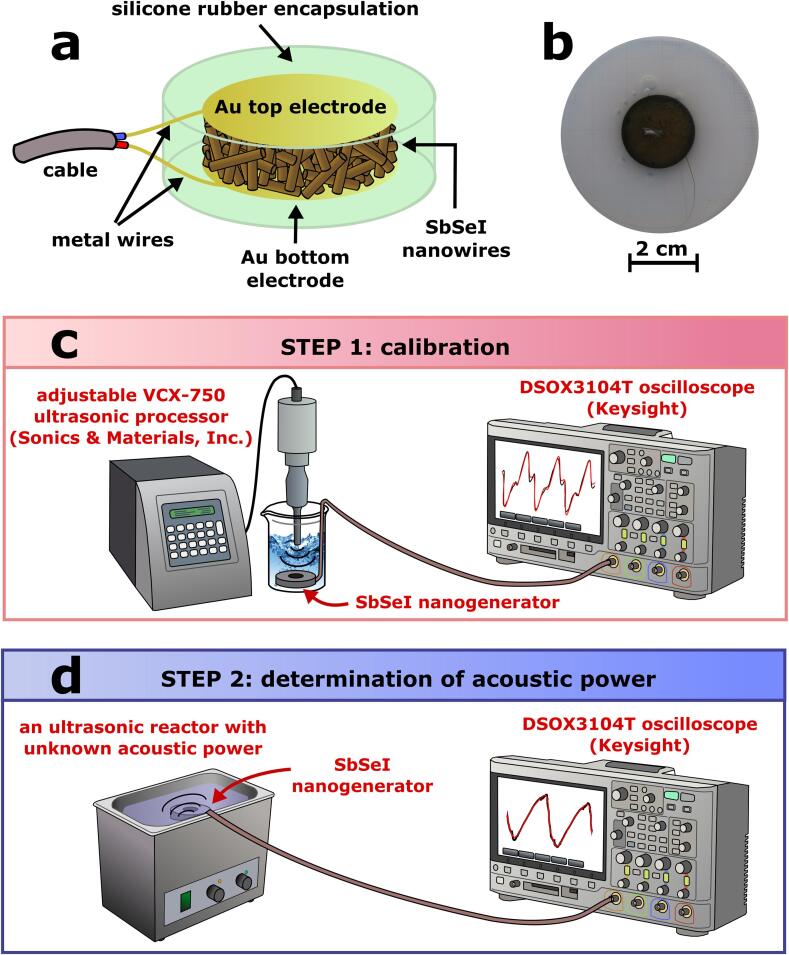
Schematic diagram (a) and photograph (b) of the prepared SbSeI nanogenerator. Schematic of the experimental measurement set up used for calibration (c) and determination of ultrasonic power (d).

The SbSeI nanogenerator fabrication process is completed using the following procedure. First, the SbSeI xerogel was processed using high pressure compression technology [35], [41], [42] that does not require a heating treatment. The material was placed into a steel cylinder, which served as a mold for the compression process. The mold was closed with a piston and mounted into a 4469 Instron testing machine. Samples were prepared in the form of a cylindrical pellet (Fig. 1a) by compressing the SbSeI xerogel at room temperature. This was completed by applying a strain of 160 MPa at 5 mm/min loading bar speed. Next, a Q150R ES rotary pump coater (Quorum Technologies Ltd.) was used to sputter gold electrodes on opposite sides of the pellet. Metal wires were attached to the sample electrodes using high purity silver paint (SPI Supplies). The sample was then covered with a protective layer to avoid any influence of water on the electric properties of the SbSeI nanogenerator. Thin films of Elastosil N10 silicone rubber (Wacker Chemie AG) were deposited on both sample surfaces. Finally, silicone rubber was completely cured after 48 h of humid atmospheric exposure. Elastosil N10 is a flexible, solvent-free, and low viscosity silicone sealant for bonding, sealing, and potting for electronic applications. According to the manufacturer, this material exhibits excellent primerless adhesion to many substrates. Note that silicone rubber is frequently used for piezoelectric hydrophone packaging [17]. A photograph of the fabricated SbSeI nanogenerator is presented in Fig. 1b.

### 2.2 Calibration of the measurement equipment

A calibration procedure was performed in the first stage, as shown in Fig. 1c. The SbSeI nanogenerator was immersed in the water bath of the VCX-750 ultrasonic reactor (Sonics & Materials, Inc.) and positioned 1 cm away from the end of the reactor sonotrode. The deionized water temperature in the bath was set to 323 K using a HAAKE DC30 thermostat with Kessel HAAKE K20 circulator (Thermo Scientific). This value corresponds to the standard temperature of a sonochemical synthesis of chalcohalide nanomaterials [35], [41], [42], [43]. Next, the nanogenerator was connected to a DSOX3104T oscilloscope (Keysight) to measure the open-circuit voltage generated under ultrasonic excitation. The ultrasound acoustic power (P_a_), emitted by a VCX-750 processor, was varied within in the range from 150 W to 750 W. According to the equipment manufacturer, the power units are rated using the RMS (root mean square) standard. Moreover, the VCX-750 reactor is capable of continuously delivering an adjusted amount of power to the irradiated liquid. The electrical response of the SbSeI nanogenerator was measured as a function of ultrasound acoustic power to determine the calibration curve. The effect of ultrasound suppression in the water bath was negligible. The attenuation coefficient was α = 4.13·10^-7^ dB/cm [44] for propagation of an ultrasonic wave with a frequency of 20 kHz in deionized water at 323 K. This means that the change in acoustic power over 1 cm is under 0.00001 %.

### 2.3 Determination of an acoustic power of ultrasounds

Fig. 1d presents the experimental setup used to measure the ultrasound acoustic power emitted by other ultrasonic reactors. The SbSeI nanogenerator was inserted into a deionized water bath at 323 K. Afterwards, the nanogenerator was connected to a DSOX3104T oscilloscope (Keysight). The electrical response to ultrasonic excitation was measured and analyzed using two different approaches. In the first, the registered waveform of the open-circuit voltage was fit to the best theoretical dependence. The acoustic power was then calculated using the fitted parameters of the electrical response and calibration curve established in the previous step. The second technique was based on the Fast Fourier Transform (FFT) of the measured electric output of the nanogenerator. Similar to the first method, a calibration dependence was used to calculate acoustic power.

## 3 Results

### 3.1 Basic characterization of the material

**Fig. 2:**
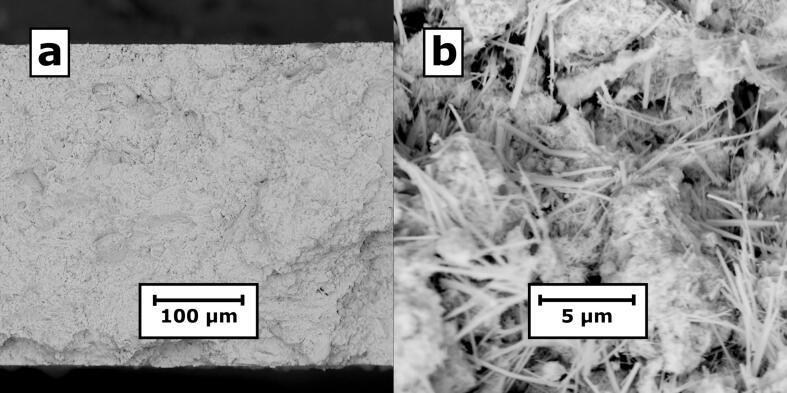
SEM micrographs of (a) cross section and (b) interior of a sample prepared by high pressure compression of SbSeI nanowires.

Fig. 2a shows SEM micrographs of the cross sectional area of a bulk sample prepared by high pressure compression of SbSeI xerogel. The nanowires are randomly oriented in the sample volume with small voids are present between them (Fig. 2b). The fill factor was determined to be 50% using sample mass, geometrical dimensions, and density of SbSeI single crystal [39].

**Fig. 3:**
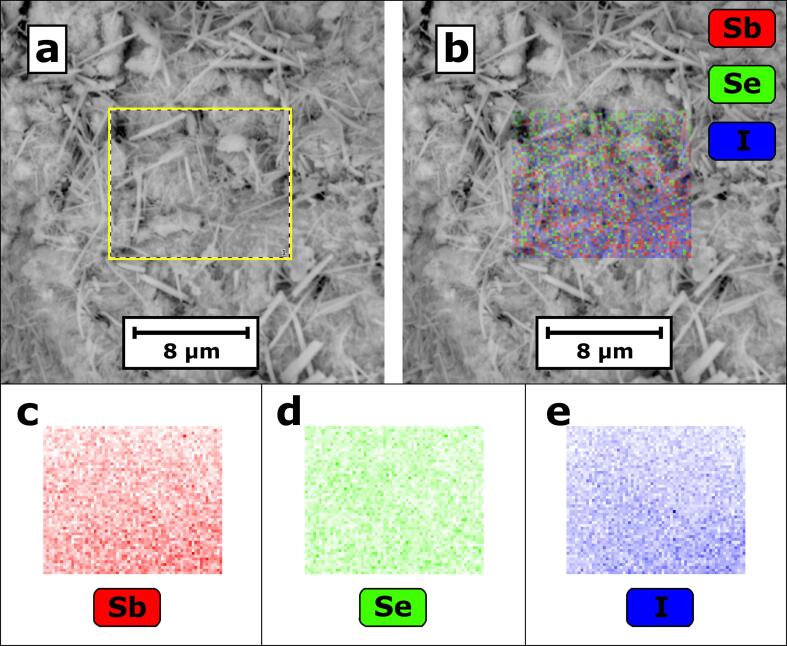
SEM image (a) of SbSeI sample interior merged with EDS elemental mapping and (b) of the area marked by the yellow rectangle in (a). Separate maps are provided showing elemental distributions of antimony (c), selenium (d), and iodine (e). (For interpretation of the references to colour in this figure legend, the reader is referred to the web version of this article.)

The chemical composition of the material was analyzed using EDS. The results confirmed that the compressed xerogel consisted of only antimony, selenium, and iodine in an elemental atomic ratio of 0.36:0.35:0.29 for Sb, Se and I averaged over the pellet volume. No other elements were detected. The EDS elemental mapping of the SbSeI xerogel is shown in Fig. 3. The antimony, selenium, and iodine are uniformly distributed in the investigated sample.

### 3.2 Best fitting of theoretical dependence to experimental data

**Fig. 4:**
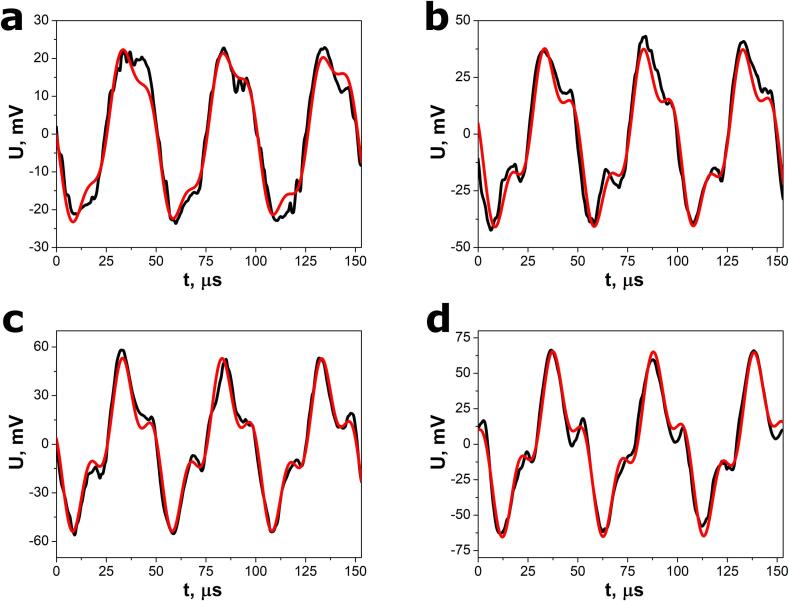
Selected open-circuit voltage responses of the SbSeI nanogenerator to ultrasound excitation for relative power (a) 20 % (b) 50 %, (c) 80 %, and (d) 100 %. The red curves represent the best fit dependence described by Eq. (1). Calibration measurements were carried out using VCX-750 (Sonics & Materials, Inc.) ultrasonic processor with ultrasound frequency f = 20 kHz and maximum power P_a_ = 750 W delivered to the sonotrode. (For interpretation of the references to colour in this figure legend, the reader is referred to the web version of this article.)

The ultrasound excitation voltage responses of the SbSeI nanogenerator measured in the calibration procedure are presented in Fig. 4. The graphs show the signals without any amplification. One can see that the amplitude of electric response rises with increasing acoustic power delivered by sonotrode to the water in the VCX-750 ultrasonic processor. The registered voltage waveforms showed the best fit to the sum of two sinusoidal functions

(1) $$U\left(t\right)=U_{1}\sin\left[2\pi f_{1}\left(t-t_{01}\right)\right]+U_{2}\sin\left[2\pi f_{2}\left(t-t_{02}\right)\right]$$

where U_1_ and U_2_ are voltage amplitudes, f_1_ and f_2_ denote frequencies, t is independent variable (time), and t_01_ and t_02_ are time constants that determine phase shift between sinusoidal signals. The best fit parameter values of Equation (1) are provided in the Table 1.

**Table 1:** The selected parameter values of Eq. (1) fit to the open-circuit voltage responses of SbSeI nanosensor shown in Fig. 4.

P_r_, %	U_1_, mV	U_2_, mV	f_1_, kHz	f_2_, kHz
20	21.07(5)	4.27(6)	19.72(1)	60.18(5)
50	32.08(6)	10.44(6)	20.070(9)	60.38(3)
80	39.13(6)	15.84(7)	19.99(1)	60.08(2)
100	47.54(7)	18.78(7)	19.701(7)	59.49(2)

**Fig. 5:**
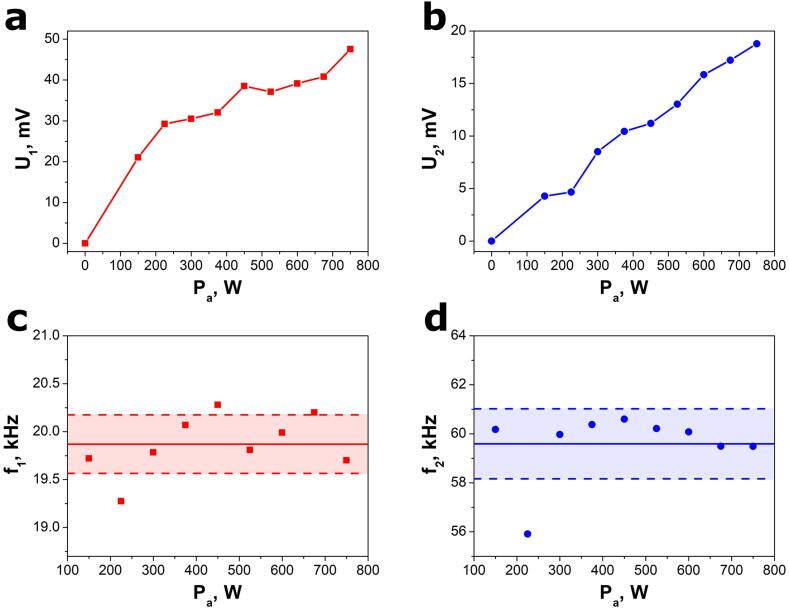
Influence of acoustic power on the selected SbSeI nanogenerator parameters: (a, b) amplitudes of open-circuit voltages and (c, d) frequencies. The horizontal solid curves in (c) and (d) represent the average values of f_1_ and f_2_, respectively. The horizontal dashed lines show the uncertainties of the calculated average values. The experiments were performed with a VCX-750 (Sonics & Materials, Inc.) ultrasonic processor.

The influence of acoustic power on the electric response parameters is shown in Fig. 5. Increasing P_a_ enhances the voltage amplitudes (U_1_ and U_2_). Simultaneously, the frequencies, f_1_ and f_2_, are independent of acoustic power within the experimental uncertainty. Therefore, average frequencies of f_1_ = 19.87(31) kHz and f_2_ = 59.6(14) kHz were measured. The f_1_ value is equal to the driving frequency of the VCX-750 reactor according to the equipment manufacturer. The f_2_ value is three times higher than f_1_ (f_2_/f_1_ = 3), meaning that f_1_ and f_2_ are fundamental and third harmonic frequencies, respectively, which is commonly observed for ultrasonic transducers [16], [45].

**Fig. 6:**
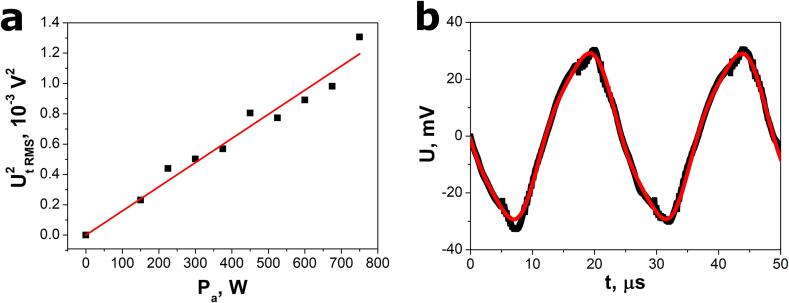
(a) Calibration curve for an electrical response of the SbSeI nanogenerator and (b) open-circuit voltage response of the SbSeI nanogenerator to ultrasound excitation emitted by a Sonic-6 reactor (Polsonic). Black points represent the experimental values. Solid red curves in (a) and (b) show the best fitted dependences described by Equations (6), (1), respectively. (For interpretation of the references to colour in this figure legend, the reader is referred to the web version of this article.)

The electrical power, generated in the SbSeI transducer under ultrasonic excitation, can be described using the following relationship [46]

(2) $$P_{e}=U_{t,RMS}^{2}∙G$$

where U_t,RMS_ is the total root mean square voltage generated in the SbSeI transducer, G = 33 µS is the electrical conductance of the nanogenerator, which was determined using an impedance analyzer 3522–50 LCR Hitester (HIOKI) at 20 kHz. The root mean square voltage for a sinusoidal waveform can be calculated using the well-known formula:

(3) $$U_{RMS}=\frac{U_{p}}{\sqrt{2}}$$

where U_p_ denotes peak (amplitude) of voltage. The voltage response of the SbSeI nanogenerator (Eq. (1)) is the sum of two sinusoidal waveforms which are orthogonal functions since f_2_/f_1_ = 3. Therefore, the total root mean square voltage is a root of the sum of all squares of the RMS components [47]

(4) $$U_{t,RMS}=\sqrt{\sum_{i=1}^{n}U_{RMS,i}^{2}}$$

where n is the number of RMS components and, in our case, n = 2. The ultrasound acoustic power is expected to be partially adsorbed by the SbSeI device and converted into electrical power described by:

(5) $$P_{e}=\gamma ∙P_{a}$$

where γ is a dimensionless coefficient related to the conversion of acoustic energy into electrical energy. Many factors influence γ, which should be dependent on ultrasonic attenuation of the silicone rubber protective layer, ultrasound absorption by the SbSeI sensing material, SbSeI nanowire piezoelectric coefficient, and the electromechanical coupling coefficient. The expression describing the SbSeI nanogenerator electrical response can be obtained by combining Equations (2), (3), (4), and (5):

(6) $$U_{t,RMS}^{2}=\frac{1}{2}\sum_{i=1}^{n}U_{i}^{2}=A∙P_{a}$$

where n is the number of sinusoidal waveforms in the nanogenerator electrical response and A = γ/G is a calibration coefficient related to device sensitivity. According to Eq. (6), the squared total RMS voltage is the linear function of acoustic power. Thus, the experimental data in Fig. 6a fit most closely with relation (6). The calibration coefficient was determined to be A = 1.59(5)·10^-6^ V^2^/W.

After the calibration procedure, the electric response of the SbSeI nanogenerator to ultrasounds emitted by a Sonic-6 reactor was measured (Fig. 6b). The registered voltage waveform showed the strongest fit to the same relation (1), which was applied during the calibration procedure. The best fit values of the parameters in Eq. (1) are listed in Table 2. The value of f_1_ corresponds to the driving frequency of Sonic-6 reactor, as claimed by the device manufacturer. Similar to the response of the SbSeI nanogenerator to ultrasound excitation emitted by the VCX-750 ultrasonic processor, the frequency of the second sinusoidal waveform is three times higher than the fundamental frequency (see Table 2). This indicates that the fundamental and third harmonic frequencies mainly contribute to the measured signal.

**Table 2:** Selected values of Eq. (1) parameters fitted to the open-circuit voltage responses of the SbSeI nanosensor presented in Fig. 6b.

U_1_, mV	U_2_, mV	f_1_, kHz	f_2_, kHz
28.40(3)	2.20(5)	40.848(3)	122.25(5)

Finally, the acoustic power of 255(8) W, emitted by a Sonic-6 reactor, was calculated using equation (6), calibration curve (Fig. 6a), and the response parameters listed in the Table 2. The conversion efficiency of 53(2) % was determined using the following relation [16]

(7) $$\eta =\frac{P_{a}}{P_{E0}}∙100\%$$

where P_E0_ = 480 W is the nominal electrical energy supplied to the ultrasonic transducer in the Sonic-6 reactor (Polsonic).

### 3.3 The method based on FFT analysis

**Fig. 7:**
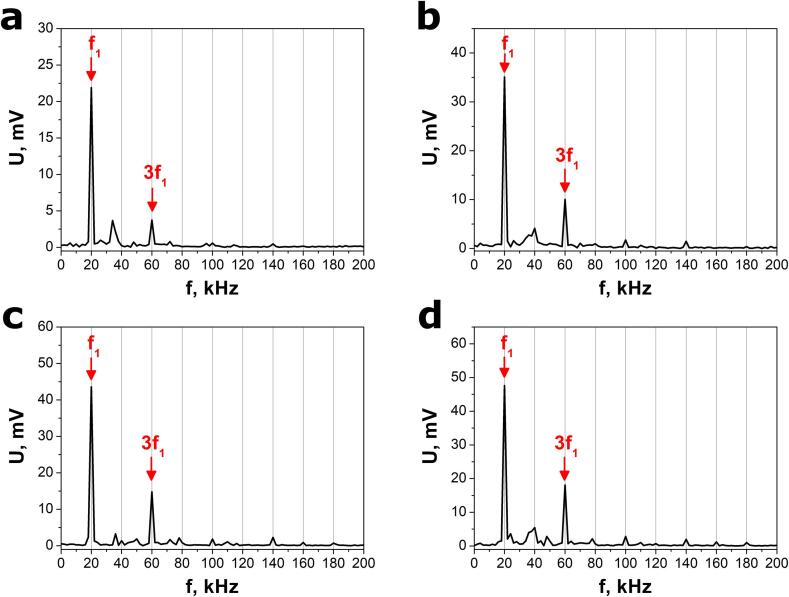
FFT spectra of select open-circuit voltage responses to ultrasound excitation with relative powers of (a) 20 % (b) 50 %, (c) 80 %, and (d) 100 %. The calibration procedure was completed by applying a VCX-750 (Sonics & Materials, Inc.) ultrasonic processor with ultrasound frequency f = 20 kHz and maximum power P_a_ = 750 W to the sonotrode.

Fig. 7 presents the FFT spectra of the open-circuit voltage response to ultrasound excitation for variable relative powers measured in the calibration procedure. These spectra reveal sharp peaks at the fundamental frequency 20 kHz, which originates from acoustic fields generated by the sonotrode and superimposed linear bubble oscillations [45]. The FFT graphs contain also small peaks corresponding to harmonics, i.e. frequencies that are integer multiples of the fundamental frequency [48]. These peaks can result from non-linear bubble oscillations [45]. However, fundamental and third harmonic frequencies primarily contribute to the registered signal. This result is in agreement with the experimental results obtained using the first method (section 3.2).

**Fig. 8:**
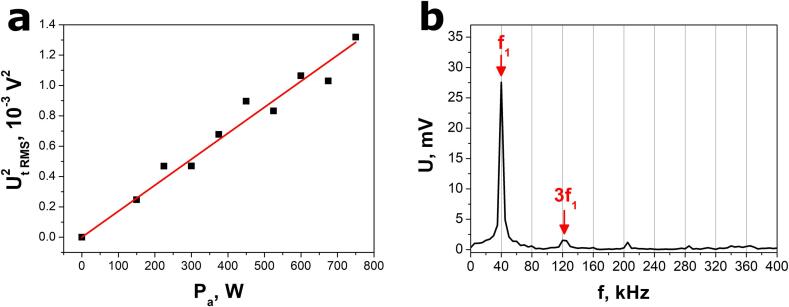
(a) Calibration curve derived from FFT analysis of an electrical response of the SbSeI nanogenerator and (b) FFT spectrum of the open-circuit voltage response to ultrasound excitation emitted by the Sonic-6 reactor (Polsonic). Black points in (a) represent experimental values of the squared total RMS voltage as a function of acoustic power. Solid red curve shows the best fit dependence (6). (For interpretation of the references to colour in this figure legend, the reader is referred to the web version of this article.)

The calibration curve (Fig. 8a) was determined by finding the best fit to the experimental data using theoretical relation (6). Only the first ten voltage amplitudes were taken into account in this procedure (n = 10). The calibration coefficient A = 1.71(5)·10^-6^ V^2^/W was calculated. Equations (4), (6) are valid since the voltage response of the SbSeI nanogenerator only consists of orthogonal sinusoidal waveforms. This is due to the fact that subsequent waveforms frequencies are multiples of the primary frequency

(8) $$f_{i}=i∙f_{1}$$

where i is the integer number.

The open-circuit voltage response to ultrasounds, emitted by the Sonic-6 reactor, was transformed to a frequency domain, as shown in Fig. 8b. The acoustic power P_a_ = 222(7) W and conversion efficiency η = 46(1) % were determined using equation (6), calibration curve (Fig. 8a), parameters of FFT spectrum (Fig. 8b), and the nominal electrical energy supplied to the ultrasonic transducer in the Sonic-6 reactor. Similar to the calibration procedure, only the first ten voltage amplitudes were taken into account for these evaluations (n = 10).

### 3.4 Examination of the device stability

**Fig. 9:**
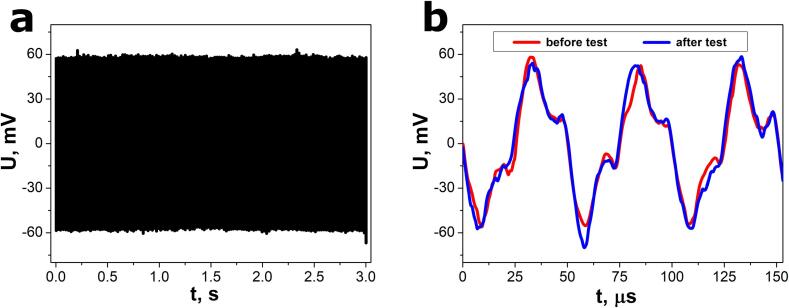
The open-circuit voltage response of the SbSeI nanogenerator for (a) 3 s and (b) 150 µs before (red curve) and after (blue curve) the device stability test. The experiment was performed using a VCX-750 (Sonics & Materials, Inc.) ultrasonic processor (f = 20 kHz; P_a_ = 600 W). (For interpretation of the references to colour in this figure legend, the reader is referred to the web version of this article.)

The electrical output of the SbSeI nanogenerator was recorded for several seconds to evaluate the device stability (Fig. 9a). The response amplitude did not change significantly during this test, which involved approximately 60,000 periods of the registered voltage waveform. The instability of ultrasound emission by the VCX-750 processor may contribute to a slight fluctuation of the voltage amplitude. The electrical output was also registered before and after the aforementioned test for a short duration of 150 µs, as presented in Fig. 9b. The measured voltage waveforms nearly overlap each other, confirming strong stability of the nanogenerator response.

### 3.5 Device tests under a low frequency mechanical excitation

**Fig. 10:**
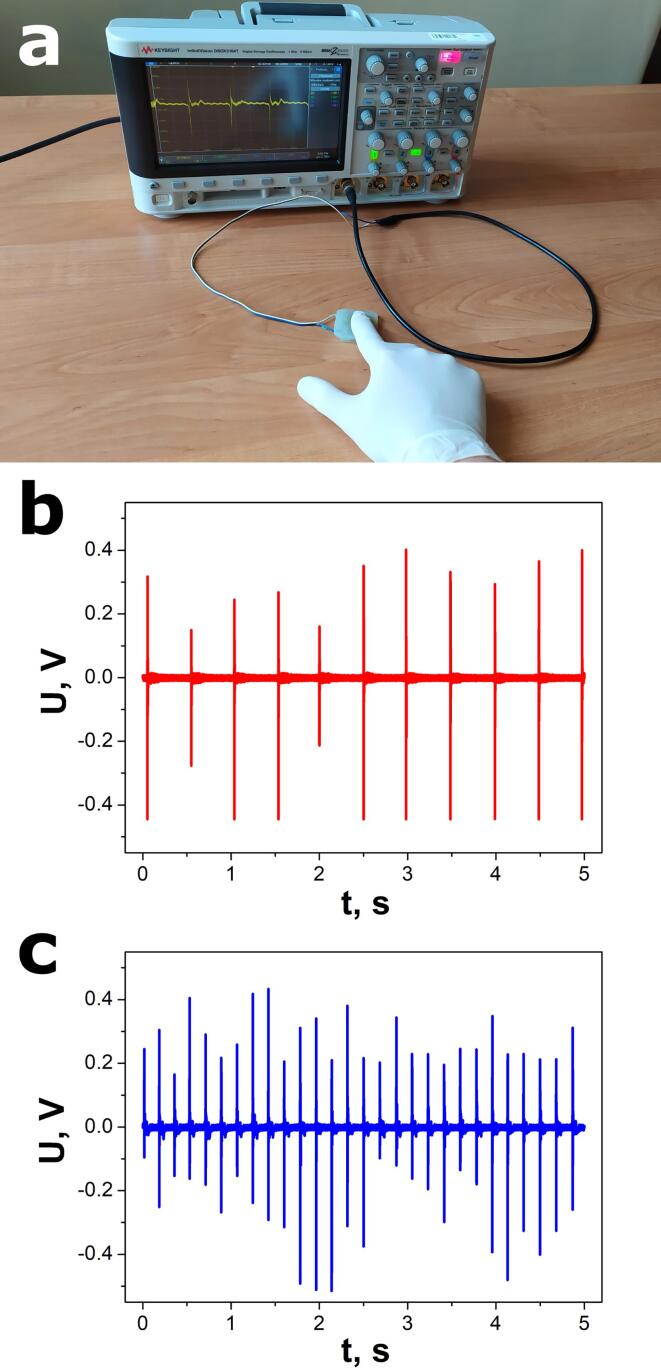
Photograph of the measurement setup used for device tests under finger tapping (a) and nanogenerator response to pressing the sample with two different frequencies f = 2.03(1) Hz (b) and f = 5.56(3) Hz (c).

A low-frequency mechanical energy is very common in our ambient environment. Similarly, human or animal motion exhibits a small frequency, typically within the 1–30 Hz range [22]. Thus, the electrical output of the developed nanogenerator was tested under mechanical excitation in the form of human finger tapping with two different frequencies (Fig. 10). Silicone gloves were used to avoid the possible influence of static charges on the measured signal. As shown in Fig. 10b and 10c, the voltage amplitude was not constant. This means that the nanogenerator electrical response was sensitive to the force applied to the device during finger tapping. The average values of the peak-to-peak voltage of 0.70(5) V and 0.55(3) V were measured for 2.03(1) Hz and 5.56(3) Hz excitation frequencies, respectively. The video file of these experiments is attached in the Supporting Information.

## 4 Discussion

The P_a_ and η values determined using the two presented techniques were compared in Table 3. These independent methods provided similar acoustic power values. The FFT method for determining acoustic power is slightly more sensitive than the alternative technique based on theoretical dependence fitting to the experimental data. This is confirmed by the higher calibration coefficient value than that determined using the previously described method. However, the FFT analysis requires much more complex computing. The uncertainties of both techniques are approximately the same.

**Table 3:** Comparison of the acoustic power and conversion efficiency values determined for a Sonic-6 reactor (Polsonic) using two different approaches.

Method	P_a_, W	η, %
Best fitting of theoretical dependence to experimental data	255(8)	53(2)
FFT analysis	222(7)	46(1)

A comprehensive overview of existing research on acoustic power sensing is provided in Table 4. The acoustic determining device presented in this paper possesses many advantages compared with other similar sensors. First, the SbSeI nanogenerator does not require an amplifier. Therefore, the experimental setup consists of only a self-powered SbSeI sensor connected to an oscilloscope, and it is easy to operate. Second, the methods proposed in this paper involve calibration of the sensor. When the measurement is performed for the same liquid as the sensor calibration, additional characterization of the liquid is not required. This is dissimilar to the classic [2], [3] and disequilibrium [4] calorimetry where the heat capacity and liquid mass must be known or measured. Furthermore, the calorimetrically determined power differs in various liquids since the measurement is affected by vapor pressure and viscosity [4]. Also, the relative uncertainty of the acoustic power measurement using an SbSeI nanogenerator is low compared with other methods such as disequilibrium calorimetry [4], pyroelectric measurements [6], [7] and optical [12] measurements. The SbSeI nanogenerator is also portable and possesses small geometrical dimensions. Its planar structure means it can be easily mounted into the ultrasonic reactor. The traditional needle-type hydrophones [49], [50] do not possess this beneficial property. They contain long probes (horns) to amplify the high-frequency longitudinal mechanical vibrations induced by alternating expansive and compressive acoustic pressure waves.

**Table 4:** A comparison of different methods and sensors used for a determination of acoustic power (u(P) means a relative uncertainty of the acoustic power measurement).

Detection method / sensor	u(P), %	Advantages	Disadvantages	Ref.
calorimetry	3 ÷ 5	simple measurement procedure	long measurement (~200 s); heat capacity and liquid mass have to be known or determined	[2], [3]
disequilibrium calorimetry	16	improved reproducibility compared with classic calorimetry	long measurement time (~600 s); heat capacity and liquid mass must be known	[4]
PVDF pyroelectric sensor	6	good repeatability	requires voltage signal filtering and amplification	[6], [7]
fiber optic probe hydrophone	5	small probe size (0.1 mm)	the need of measured signal amplification; sophisticated measurement setup	[12]
electrical impedance measurements	0.7 ÷ 7.1	low cost	requires extensive knowledge of transducer parameters, requires amplifier application	[16]
PZT piezoelectric hydrophone		small sensor dimensions	complex fabrication process of the sensor; complicated calculations; requires an amplifier; high sensor noise	[17]
anechoic tank with hydrophone			problems determining the equivalent diameter of the ultrasounds source (sonotrode tip with bubble cloud)	[51]
SbSeI piezoelectric nanogenerator	3.1	measurement simplicity; no amplifier requirement; small sensor size; facile sensor fabrication	calibration of the sensor required	this paper

Conventional methods for piezoelectric ceramic fabrication [52] rely on high temperature treatment, including calcination and sintering. This is a significant disadvantage comparison with SbSeI nanogenerator fabrication, which is performed successfully at room temperature. Furthermore, the nanogenerator dimensions are dependent on the applied mold during the compression process. This provides easy tunability regarding the size of the device. When an optimal geometry is achieved, the resonant frequency of the sample corresponds to the excitation frequency of ultrasounds, enhancing device sensitivity.

The acoustic impedance Z is the measure of the opposition that a system presents to acoustic flow resulting from applied acoustic pressure [53]. This is defined by the following equation:

(9) Z=ρ∙c

where ρ is density of the medium and c is the sound wave speed in the medium. The acoustic power, dissipated in the liquid, is proportional to the acoustic impedance of the liquid [5]:

(10) $$P_{a}=\frac{1}{2}A^{2}{\left(2\pi f\right)}^{2}S∙Z$$

where A is the amplitude of the ultrasonic wave, f is frequency, and S is the emitter area of the horn or sonotrode. Based on Equation (10), even if the A, f, and S parameters are constant, the acoustic power can differ for various liquids. However, this problem is easily solved using the technology presented in this paper. The acoustic impedance of the liquid does not need to be known if the measurement and calibration are performed using the same fluid. This means that further evaluation of liquid density and ultrasonic wave speed is not required. This is a great advantage for using this technology in sonochemistry where complex chemical composition solutions are frequently applied.

Antimony selenoiodide exhibits both semiconducting and piezoelectric properties. Thus, temperature influences its electrical properties. Similarly, temperature change should result in sensitivity variation in the SbSeI nanogenerator. A piezoelectric coupling coefficient usually decreases with increasing temperature, leading to the reduction in response of the piezoelectric harvester [54]. In our case, this effect was eliminated by using a thermostat that maintained a constant water bath temperature in the ultrasonic reactor. However, the influence of temperature on the SbSeI nanogenerator response will be investigated in a near future.

## 5 Conclusions

The presented methods to determine acoustic power are fast, which is significant compared with other known techniques. The rapid measurement, performed during acquisition times less than 1 ms, enables real time monitoring of ultrasonic reactor operation. This goal cannot be achieved using thermoelectric or pyroelectric sensors, which have response times on the order of tens of minutes. In addition, the SbSeI nanogenerator is a portable, small, self-powered device with a planar structure. Thus, it may be readily mounted into the ultrasonic reactor. This feature is a great advantage over traditional needle-type hydrophones with inconvenient geometry. The fabrication technology of the SbSeI nanogenerator is scalable and easy to optimize in order to obtain the most sensitive devices. Moreover, the nanogenerator fabrication process does not involve annealing or sintering, which is commonly used for hydrophones made from piezoelectric ceramics.

The determination of acoustic power using FFT was found to be slightly more sensitive than the technique based on the theoretical dependence fitting to the experimental data. This was confirmed by a higher value for the calibration coefficient. However, this benefit is accompanied by more complex computing. The uncertainties of both methods did not differ significantly. It should be emphasized that the results of the two independent approaches are convergent. The acoustic powers and conversion efficiencies, determined using the two methods described in this paper, were very close to each other.

The fundamental and third harmonics were recognized as frequencies that contribute mainly to the signal of the SbSeI nanogenerator under ultrasonic excitation. This effect is usually observed in ultrasonic transducers. Analysis of the nanogenerator response in the range of MHz frequencies would allow for additional study of this acoustic cavitation noise. Such investigations will be performed in the near future.

## CRediT authorship contribution statement

**Krystian Mistewicz:** Conceptualization, Methodology, Validation, Formal analysis, Investigation, Supervision, Funding acquisition, Writing - original draft. **Marcin Jesionek:** Investigation. **Hoe Joon Kim:** Writing - review & editing. **Sugato Hajra:** Writing - review & editing. **Mateusz Kozioł:** Investigation. **Łukasz Chrobok:** Investigation. **Xudong Wang:** Supervision, Writing - review & editing.

## Declaration of Competing Interest

The authors declare that they have no known competing financial interests or personal relationships that could have appeared to influence the work reported in this paper.
